# Supplementary material for: Dietary Patterns in Pregnancy and the Postpartum Period and the Relationship with Maternal Weight up to One Year after Pregnancy Complicated by Gestational Diabetes
Source: Nutrients. 2023 Oct 5;15(19):4258. doi: 10.3390/nu15194258 (PMC10574553; doi:10.3390/nu15194258)
Supplement: Supplementary file 1 [file nutrients-15-04258-s001.zip › nutrients-2553159-supplementary.pdf]

nutrients

Table S1. Adjusted associations * between food items with the highest factor loading of each dietary pattern identified during pregnancy and postpartum BMI modification ** in women with pregnancy complicated by gestational diabetes mellitus. LINDA-Brasil Study.

	BMI at six months postpartum			BMI at twelve months postpartum		
	**RR**	**[95% CI]**	**P-value**	**RR**	**[95% CI]**	**P-value**
			**Pregnancy**			
**Risk pattern**						
Fried foods						
**Model 1**						
0-1/week						
2-4/week	0.93	[0.75 - 1.15]	0.511	0.96	[0.77- 1.19]	0.733
5-7/week	1.61	[1.25 - 2.07]	<0.001	1.47	[1.08- 2.02]	0.015
**Model 2**						
0-1/week						
2-4/week	1.01	[0.82 - 1.25]	0.899	1.04	[0.84- 1.29]	0.709
5-7/week	1.83	[1.42 - 2.37]	<0.001	1.48	[1.06- 2.07]	0.022
**Model 3**						
0-1/week						
2-4/week	1.01	[0.82 - 1.25]	0.928	1.04	[0.84- 1.29]	0.694
5-7/week	1.83	[1.43 - 2.34]	<0.001	1.48	[1.06- 2.07]	0.023
Cookies and sweets						
**Model 1**						
0-1/week						
2-4/week	1.01	[0.84- 1.22]	0.870	0.89	[0.73- 1.09]	0.276
5-7/week	1.03	[0.81- 1.31]	0.804	0.87	[0.66- 1.13]	0.292
**Model 2**						
0-1/week						
2-4/week	1.08	[0.90 - 1.30]	0.389	0.94	[0.77- 1.15]	0.546
5-7/week	1.16	[0.91- 1.48]	0.236	0.92	[0.70- 1.21]	0.550
**Model 3**						
0-1/week						
2-4/week	1.07	[0.89- 1.28]	0.477	0.93	[0.76- 1.13]	0.460
5-7/week	1.15	[0.90- 1.47]	0.254	0.92	[0.70- 1.20]	0.536
Sweetened beverages						
**Model 1**						
0-1/week						
2-4/week	1.05	[0.86- 1.29]	0.618	0.84	[0.66- 1.06]	0.151
5-7/week	1.24	[1.03- 1.49]	0.022	1.24	[1.03- 1.48]	0.019
**Model 2**						
0-1/week						
2-4/week	1.09	[0.89- 1.33]	0.395	0.85	[0.68- 1.08]	0.194
5-7/week	1.33	[1.10- 1.60	0.003	1.30	[1.08- 1.55]	0.005
**Model 3**						
0-1/week						
2-4/week	1.09	[0.89- 1.33]	0.400	0.86	[0.68- 1.09]	0.217
5-7/week	1.31	[1.09- 1.58]	0.004	1.29	[1.08- 1.55]	0.005
Processed meat						

**Model 1**								
0-1/week								
2-4/week	1.06	[0.88-	1.27]	0.529	0.97	[0.80-	1.17]	0.746
5-7/week	1.20	[0.97-	1.48]	0.074	1.07	[0.85-	1.33]	0.581
**Model 2**								
0-1/week								
2-4/week	1.05	[0.87-	1.26]	0.571	0.94	[0.78-	1.14]	0.554
5-7/week	1.20	[0.97-	1.48]	0.094	1.07	[0.85-	1.34]	0.531
**Model 3**								
0-1/week								
2-4/week	1.04	[0.87-	1.25]	0.625	0.94	[0.77-	1.13]	0.506
5-7/week	1.18	[0.95-	1.45]	0.122	1.07	[0.85-	1.33]	0.564
**Healthy pattern**								
Red meat without visible fat								
**Model 1**								
0-1/week								
2-4/week	0.84	[0.70-	1.00]	0.005	0.91	[0.75-	1.10]	0.346
5-7/week	1.15	[0.94-	1.40]	0.170	1.16	[0.94-	1.43]	0.153
**Model 2**								
0-1/week								
2-4/week	0.83	[0.69-	1.00)	0.054	0.89	[0.74-	1.08]	0.240
5-7/week	1.16	[0.95-	1.41]	0.149	1.15	[0.94-	1.42]	0.166
**Model 3**								
0-1/week								
2-4/week	0.84	[0.70-	1.00]	0.057	0.89	[0.74-	1.08]	0.247
5-7/week	1.15	[0.95-	1.41]	0.156	1.15	[0.94-	1.42]	0.158
Raw salad								
**Model 1**								
0-1/week								
2-4/week	0.89	[0.70-	1.12]	0.322	0.74	[0.57-	0.95]	0.019
5-7/week	0.82	[0.65-	1.03]	0.094	0.87	[0.69-	1.09]	0.239
**Model 2**								
0-1/week								
2-4/week	0.85	[0.67-	1.08]	0.179	0.73	[0.57-	0.94]	0.014
5-7/week	0.77	[0.61-	0.97]	0.027	0.84	[0.67-	1.05]	0.126
**Model 3**								
0-1/week								
2-4/week	0.85	[0.67-	1.07]	0.176	0.73	[0.56-	0.93]	0.012
5-7/week	0.77	[0.62-	0.97]	0.029	0.84	[0.67-	1.05]	0.129
Cooked salad								
**Model 1**								
0-1/week								
2-4/week	1.00	[0.84-	1.20]	0.987	0.92	[0.76-	1.11]	0.387
5-7/week	0.78	[0.61-	0.99]	0.048	0.77	[0.60-	0.98]	0.039
**Model 2**								
0-1/week								
2-4/week	0.99	[0.82-	1.18]	0.909	0.91	[0.75-	1.09]	0.315
5-7/week	0.75	[0.59-	0.96]	0.026	0.75	[0.59-	0.95]	0.020
**Model 3**								
0-1/week								
2-4/week	0.99	[0.83-	1.19]	0.992	0.91	[0.75-	1.09]	0.298
5-7/week	0.76	[0.59-	0.97]	0.029	0.75	[0.59-	0.95]	0.019

**DASH**						
Low fat dairy						
**Model 1**						
0-1/week						
2-4/week	0.94	[0.70- 1.28]	0.714	1.08	[0.83- 1.40]	0.564
5-7/week	1.12	[0.95- 1.32]	0.158	0.99	[0.84- 1.18]	0.964
**Model 2**						
0-1/week						
2-4/week	0.93	[0.68- 1.26]	0.642	1.09	[0.83- 1.42]	0.533
5-7/week	1.09	[0.92- 1.28]	0.309	0.97	[0.81- 1.15]	0.722
**Model 3**						
0-1/week						
2-4/week	0.95	[0.71- 1.30]	0.769	0.98	[0.78- 1.24]	0.915
5-7/week	1.08	[0.92- 1.27]	0.337	0.95	[0.56- 1.57]	0.847

* Poisson regressions with robust variance adjusted for the outcome; ** increase in BMI between 2 months and 6 months to 12 months post-partum and frequency of exposure of dietary items. Model 1: Adjusted for age, skin color, income, city, smoking during pregnancy and number of previous pregnancies. Model 2: Adjusted for model 1 + pre-pregnancy BMI and Gestational weight gain. Model 3: Adjusted for model 2 + Breastfeeding in the follow-up period.

Table S2. Adjusted associations * between food items with the highest factor loading of each dietary pattern identified six months after delivery and postpartum BMI modification ** in women with pregnancy complicated by gestational diabetes mellitus. LINDA-Brasil Study.

	**BMI at six months postpartum**			**BMI at twelve months postpartum**		
	**RR**	**[95% CI]**	**P-value**	**RR**	**[95% CI]**	**P-value**
			**Postpartum**			
**Risk pattern**						
Sweetened beverages						
**Model 1**						
0-1/week						
2-4/week	1.14	[0.93- 1.40]	0.213	1.06	[0.85- 1.32]	0.610
5-7/week	1.09	[0.89- 1.34]	0.386	1.24	[1.01- 1.52]	0.015
**Model 2**						
0-1/week						
2-4/week	1.18	[0.96- 1.44]	0.109	1.08	[0.86- 1.35]	0.516
5-7/week	1.16	[0.95- 1.42]	0.147	1.27	[1.04- 1.55]	0.017
**Model 3**						
0-1/week						
2-4/week	1.17	[0.95- 1.43]	0.136	1.06	[0.85- 1.33]	0.583
5-7/week	1.16	[0.95- 1.41]	0.154	1.27	[1.04- 1.55]	0.019
Fried foods						
**Model 1**						
0-1/week						
2-4/week	1.08	[0.89- 1.31]	0.428	0.92	[0.73- 1.16]	0.487
5-7/week	1.63	[1.15- 2.31]	0.006	1.42	[0.90- 2.24]	0.126
**Model 2**						
0-1/week						
2-4/week	1.07	[0.88- 1.29]	0.507	0.87	[0.70- 1.09]	0.234
5-7/week	1.85	[1.39- 2.47]	<0.001	1.50	[1.01- 2.23]	0.043
**Model 3**						
0-1/week						

2-4/week	1.05	[0.86-	1.27]	0.645	0.86	[0.68-	1.08]	0.189
5-7/week	1.87	[1.40;	2.50)	<0.001	1.52	[1.00-	2.29]	0.047
Processed meat								
**Model 1**								
0-1/week								
2-4/week	1.12	[0.95-	1.32]	0.185	0.99	[0.82-	1.19]	0.912
5-7/week	0.95	[0.72-	1.25]	0.712	1.18	[0.93-	1.50]	0.179
**Model 2**								
0-1/week								
2-4/week	1.12	[0.95-	1.33]	0.173	0.98	[0.81-	1.17]	0.810
5-7/week	0.93	[0.71-	1.21]	0.580	1.15	[0.91-	1.47]	0.242
**Model 3**								
0-1/week								
2-4/week	1.11	[0.94-	1.32]	0.195	0.97	[0.81-	1.17]	0.774
5-7/week	0.90	[0.70-	1.18]	0.451	1.13	[0.89-	1.44]	0.307
Cookies and sweets								
**Model 1**								
0-1/week								
2-4/week	1.11	[0.93-	1.32]	0.227	1.09	[0.92-	1.30]	0.313
5-7/week	1.36	[1.08-	1.70]	0.008	0.98	[0.74-	1.31]	0.910
**Model 2**								
0-1/week								
2-4/week	1.16	[0.98-	1.37]	0.082	1.18	[0.94-	1.33]	0.209
5-7/week	1.44	[1.14-	1.82]	0.002	0.97	(0.73-	1.29]	0.850
**Model 3**								
0-1/week								
2-4/week	1.16	[0.98-	1.37]	0.091	1.11	[0.94-	1.33]	0.211
5-7/week	1.44	[1.14-	1.82]	0.002	0.97	[0.73-	1.30]	0.860
**Mixed pattern**								
Natural fruit juice								
**Model 1**								
0-1/week								
2-4/week	1.11	[0.91-	1.36]	0.290	0.98	[0.79-	1.22]	0.879
5-7/week	0.92	[0.71-	1.19]	0.547	0.87	[0.66-	1.15]	0.339
**Model 2**								
0-1/week								
2-4/week	1.09	[0.90-	1.33]	0.375	0.96	[0.78-	1.19]	0.742
5-7/week	0.90	[0.70-	1.17]	0.444	0.87	[0.66-	1.15]	0.343
**Model 3**								
0-1/week								
2-4/week	1.08	[0.89-	1.32]	0.417	0.96	[0.77-	1.18]	0.687
5-7/week	0.91	[0.70;	1.17)	0.463	0.87	[0.66-	1.15]	0.329
Salty snacks								
**Model 1**								
0-1/week								
2-4/week	1.00	[0.84-	1.19]	0.946	1.07	[0.90-	1.28]	0.454
5-7/week	1.00	[0.80-	1.26]	0.994	1.20	[0.96-	1.49]	0.098
**Model 2**								
0-1/week								
2-4/week	0.98	[0.83-	1.17]	0.880	1.06	[0.89-	1.27]	0.506
5-7/week	0.97	[0.77-	1.22]	0.782	1.13	[0.90-	1.40]	0.284

**Model 3**								
0-1/week								
2-4/week	0.98	[0.83-	1.16]	0.866	1.07	[0.89-	1.27]	0.464
5-7/week	0.94	[0.75-	1.19]	0.638	1.11	[0.89-	1.39]	0.348
Fruits								
**Model 1**								
0-1/week								
2-4/week	0.89	[0.73-	1.08]	0.242	0.81	[0.66-	0.98]	0.032
5-7/week	0.87	[0.72-	1.08)	0.176	0.76	[0.63-	0.93]	0.0078
**Model 2**								
0-1/week								
2-4/week	0.86	[0.71-	1.05]	0.147	0.79	[0.65-	0.96]	0.020
5-7/week	0.84	[0.69-	1.03)	0.090	0.75	[0.61-	0.91]	0.004
**Model 3**								
0-1/week								
2-4/week	0.87	[0.71-	1.06]	0.166	0.79	[0.65-	0.96]	0.021
5-7/week	0.86	[0.72-	1.05]	0.128	0.76	[0.62-	0.92]	0.006
Chicken								
**Model 1**								
0-1/week								
2-4/week	0.82	[0.66-	1.03]	0.092	1.01	[0.76-	1.35]	0.912
5-7/week	0.75	[0.58-	0.98]	0.037	1.00	[0.73-	1.36]	0.996
**Model 2**								
0-1/week								
2-4/week	0.78	[0.62-	0.97]	0.031	0.97	[0.74-	1.28]	0.844
5-7/week	0.70	[0.54-	0.91]	0.008	0.96	[0.70-	1.30]	0.781
**Model 3**								
0-1/week								
2-4/week	0.79	[0.63-	0.98]	0.037	0.97	[0.74-	1.28]	0.857
5-7/week	0.70	[0.53-	0.91]	0.008	0.95	[0.70-	1.29]	0.744
**DASH Type**								
Cooked salad								
**Model 1**								
0-1/week								
2-4/week	0.94	[0.78-	1.12]	0.473	0.83	[0.69-	0.98]	0.037
5-7/week	0.97	[0.76-	1.23]	0.784	0.81	[0.63-	1.05]	0.115
**Model 2**								
0-1/week								
2-4/week	0.91	[0.77-	1.09]	0.315	0.82	[0.69-	0.98]	0.030
5-7/week	0.96	[0.76-	1.22]	0.770	0.83	[0.65-	1.07]	0.149
**Model 3**								
0-1/week								
2-4/week	0.92	[0.77-	1.09]	0.333	0.83	[0.69-	0.98]	0.034
5-7/week	0.99	[0.78-	1.25]	0.928	0.85	[0.66-	1.09]	0.209
Raw salad								
**Model 1**								
0-1/week								
2-4/week	0.90	[0.74-	1.11]	0.333	0.85	[0.69-	1.03]	0.099
5-7/week	0.90	[0.73-	1.10]	0.327	0.73	[0.59-	0.89]	0.003
**Model 2**								
0-1/week								

2-4/week	0.85	[0.70-	1.05]	0.131	0.80	[0.66-	0.97]	0.026
5-7/week	0.85	[0.69-	1.04]	0.118	0.68	[0.55-	0.84]	<0.001
**Model 3**								
0-1/week								
2-4/week	0.85	[0.69-	1.05]	0.132	0.80	[0.65-	0.97]	0.024
5-7/week	0.84	[0.68-	1.04]	0.115	0.68	[0.55-	0.84]	<0.001
Low fat dairy								
**Model 1**								
0-1/week								
2-4/week	1.07	[0.85-	1.34]	0566	0.99	[0.78-	1.26]	0.960
5-7/week	0.85	[0.46-	1.54]	0.605	0.97	[0.62-	1.54]	0.916
**Model 2**								
0-1/week								
2-4/week	1.07	[0.85-	1.34]	0.980	0.98	[0.78-	1.24]	0.910
5-7/week	0.78	[0.40-	1.52]	0.476	0.93	[0.56-	1.54]	0.790
**Model 3**								
0-1/week								
2-4/week	1.07	[0.85-	1.33)	0.555	0.99	[0.78-	1.24]	0.915
5-7/week	0.81	[0.42-	1.58)	0.538	0.95	[0.58-	1.57]	0.847

* Poisson regressions with robust variance adjusted for the outcome increase in. ** BMI between 2 months and 6 months to 12 months post-partum and frequency of exposure of dietary items. Model 1: Adjusted for age, skin color, income, city, smoking during pregnancy and number of previous pregnancies. Model 2: Adjusted for model 1 + pre-pregnancy BMI and Gestational weight gain. Model 3: Adjusted for model 2 + + Breastfeeding in the follow-up period.
